# A mouthwash formulated with o-cymen-5-ol and zinc chloride specifically targets potential pathogens without impairing the native oral microbiome in healthy individuals

**DOI:** 10.1080/20002297.2023.2185962

**Published:** 2023-03-03

**Authors:** Javier Pascual, Javier Mira Otal, Daniel Torrent-Silla, Manuel Porcar, Cristina Vilanova, Fernando Vivancos Cuadras

**Affiliations:** a Darwin Bioprospecting Excellence S.L., Paterna, Spain; b Lacer S. A., Barcelona, Spain; c Institute for Integrative Systems Biology I2SysBio (University of Valencia - CSIC), Paterna, Spain

**Keywords:** Mouthwash, cymen-5-ol, zinc chloride, pathogens, oral microbiome

## Abstract

**Background:**

Many antimicrobial compounds in mouthwashes can have a negative impact on the oral microbiome. O-cymen-5-ol, a compound derived from a phytochemical, has a targeted mode of action and is being used as an alternative. However, its effect on the native oral microbiome is unknown.

**Aim:**

To assess the effect of a mouthwash formulated with o-cymen-5-ol and zinc chloride on the oral microbiome of healthy individuals.

**Methods:**

A mouthwash formulated with o-cymen-5-ol and zinc chloride was administered to a cohort of 51 volunteers for 14 days, while another cohort of 49 volunteers received a placebo. The evolution of the oral microbiome in both groups was analysed using a metataxonomic approach.

**Results:**

Analysis of the oral microbiome showed that the mouthwash selectively targeted potential oral pathogens while maintaining the integrity of the rest of the microbiome. Specifically, the relative abundance of several potentially pathogenic bacterial taxa, namely *Fusobacteriota*, *Prevotella*, *Actinomyces*, *Granulicatella*, *Abiotrophia*, *Lautropia*, *Lachnoanaerobaculum*, *Eubacterium* (nodatum group) and *Absconditabacteriales* (SR1) decreased, while the growth of *Rothia*, a nitrate-reducing bacterium beneficial for blood pressure, was stimulated.

**Conclusions:**

The use of o-cymen-5-ol and zinc chloride as antimicrobial agents in oral mouthwashes is a valuable alternative to classical antimicrobial agents.

## Introduction

A healthy human oral cavity harbours one of the most complex and diverse human microbiomes comprising over 10^9^ bacteria belonging to at least 700 different species [1,2]. Besides bacteria, the community harbours other microorganisms such as fungi, viruses, archaea and protozoa, which have key roles in shaping the oral ecosystem [1–5]. Under healthy conditions, microorganisms inhabiting the oral cavity are in a state of ecological equilibrium, and they are involved in many pivotal functions for maintaining the oral and systemic health of the host [6,7]. Most of the oral bacteria are commensals [8], some of which carry out beneficial roles by preventing the proliferation of potential pathogenic microorganisms [9]. However, a disruption in the balance of the healthy oral microbiome can occur because of intrinsic and extrinsic factors such as dietary changes, antibiotic treatment, immune diseases, tissue damage, and even due to the use of mouthwashes containing antimicrobial agents with large spectrum and unspecified mode of action [10–12]. This dysbiosis can lead to the emergence of oral diseases such as gum diseases (gingivitis and periodontitis) and tooth decay (caries) [10,13,14].

Mouthwashes are used by oral health care practitioners, as well as consumers with and without oral diseases, to reduce bacterial load within the oral cavity on the pretext of preventing and managing oral diseases [15]. More specifically, mouthwashes are used to inhibit the colonisation of harmful microorganisms, reduce the formation of dental plaque, and prevent gingival bleeding caused by sensitive gums and gingivitis. The most frequently used antimicrobial agents are of synthetic origin, notably chlorhexidine (CHX), cetylpyridinium chloride and triclosan, and are characterised by a broad spectrum of action against microorganisms, often triggering major changes in native oral microbiomes. In addition, some of these compounds can cause toxicity problems and contribute to making bacteria resistant to antibiotics [16–19]. For example, the US Food & Drug Administration (FDA) has banned the use of triclosan in certain antiseptic wash products [20], while it is still allowed in others, such as toothpastes and mouthwashes, but is currently under review [21].

In recent years, the use of plant-derived biomolecules in oral hygiene products has increased, driven by the emergence of multidrug-resistant pathogens and the need for economical, safe and effective substitutes [16,22]. Essential oils such as eucalyptol, menthol, methyl salicylate and thymol are recognised in the FDA monograph to deliver antigingivitis efficacy in mouthwash [23], and they have also been reported to be effective in toothpaste against gingivitis [24]. However, thymol in particular causes a rather polarising, burning sensation, which limits acceptability for family-orientated oral care products [25]. The o-cymen-5-ol (C_10_H_14_O) is a substituted phenolic compound derivative of isopropyl cresol. It was first synthesized in 1954 as an isomer of thymol, and it is colorless, odorless, and it has a high stability and a favourable safety profile [26]. To date, several studies have shown that its use in combination with zinc chloride prevents the proliferation of pathogenic bacteria and, therefore, the appearance of the aforementioned oral pathologies, as well as halitosis, gingival bleeding, plaque formation and dental erosion [25,27,28]. However, the direct effect of the mix o-cymen-5-ol/zinc salts on the native oral microbiome, not only against potential pathogenic microorganisms but also against commensal bacteria, is unknown to date.

The aim of this study was to analyse the effects of a mouthwash formulated with o-cymen-5-ol and zinc chloride on the oral microbiome in the subgingival area of healthy individuals, in order to determine whether or not it disrupts the structure of the native microbiome, specifically inhibiting potentially pathogenic microorganisms and/or promoting beneficial microorganisms. Our results confirm that the new mouthwash does not generate a dysbiosis in the commensal bacterial community and, in turn, decreases the abundance of several bacteria associated with oral diseases. It also promoted the presence of a nitrate-reducing bacterium, which has been shown to be beneficial for oral health and blood pressure. These results pave the way for the development of new mouthwashes based on plant-derived bioactive compounds to prevent and treat oral diseases such as dental plaque and gingival bleeding caused by sensitive gums and gingivitis.

## Materials and methods

### Subject recruitment and trial with mouthwashes

The recruitment of subjects and the collection of biological samples were carried out by Zurko Research S.L. (Spain) under the supervision of a pharmacist and a dentist. Initially, the cohort comprised 105 volunteers, although five volunteers withdrew voluntarily during the experimental phase. Therefore, the bioinformatic and statistical studies were based on a cohort of 100 healthy volunteers of both genders and aged 18–65 years (Table 1; Table S1). Volunteers were evaluated for oral health, including the use of a standard periodontal exam, with spot probing for bleeding and loss of attachment, and an oral health subject history. Exclusion criteria are detailed in Supplementary Material. The study was conducted following the general conditions of Zurko Research S.L. established for the execution of human study projects (Structure and Content of Clinical Study Reports from ICH Harmonised Tripartite Guideline; Good Clinical Practice Guideline E6 (R2) of 14 June 2017, EMA/CHMP/ICH/135/1995 of 1 May 1996, Guideline 2001/20/EC of the European Parliament and of the Council − 1 May 2001). All participants were previously informed about the type and procedures of the study by signing an Informed Consent form before starting the study.

**Table 1.** Demographics information of volunteers. The mouthwash formulated with o-cymen-5-ol and zinc chloride was tested on 49 individuals, whereas 51 volunteers received a placebo treatment. The subgingival microbiome of each volunteer was analysed at the beginning and at the end of the experiment after 14 days.

	Mouthwash (*n* = 49)	Placebo (*n* = 51)
Gender	Female = 35	Female = 36
	Male = 14	Male = 15
Age	18–30 = 20	18–30 = 16
	31–40 = 15	31–40 = 18
	41–50 = 11	41–50 = 9
	50–68 = 3	50–68 = 8
Toothpaste	Binaca = 1	Alipide = 1
	Colgate = 39	Bonté = 1
	Lacer = 1	Colgate = 32
	Marvis = 1	Deliplus = 5
	Oral B = 1	Gingilacer = 1
	Parodontax = 2	Licor del polo = 3
	Sensodine = 1	Oral B = 5
	Signal = 2	Parodontax = 1
	Vitis = 1	Sensodine = 1
		Vitis = 1

### Treatment with the mouthwashes and collection of subgingival samples

A double-blind, randomized study was conducted based on two study groups, one treated with the mouthwash formulated with the o-cymen-5-ol/zinc system (*n* = 49) and the other treated with a placebo based on sterile distilled water (*n* = 51). The mouthwash was prepared with o-cymen-5-ol (0.1%) in combination with a zinc salt (0.1%) because of its synergistic effect in inhibiting oral pathogens [25]. The complete formulation of the mouthwash is detailed in Table S2. The daily oral hygiene procedure was standardised among the volunteers as well as the collection of the biological samples. The administration schedule consisted of two daily one-minute rinses (after lunch and dinner, always after brushing their teeth) of 10 ml of the mouthwash or placebo, depending on the assigned group, for a total period of 14 days. Each volunteer used the toothpaste they normally use (Table S1). To avoid bias, the mouthwash and placebo were given to the volunteers in the same opaque bottles. Two samples were taken from the subgingival area of each volunteer, one before starting treatment (T0) and one at the end of the study after 14 days (T14). Sampling was conducted in the morning, without participants having ingested any food or drink (except water) and without having brushed their teeth or used the mouthwash/placebo since the previous night. Sterile swabs were used to collect the biological samples. The subgingival area was sampled in four areas of the mouth: upper right, upper left, lower right, and lower left quadrants. Once sampled, the swabs were placed in cryotubes containing a DNA stabilising solution and then preserved at −20°C until analysis in the laboratory.

### Taxonomic composition of the subgingival microbiome

Taxonomic composition of the subgingival microbiome was studied by next-generation sequencing (NGS) of 16S rRNA amplicons. Metagenomic DNA was extracted using the DNeasy PowerSoil Pro Kit (QIAGEN, Hilden, Germany), and the extracted DNA was quantified using the QUBIT dsDNA HS-High Sensitivity Kit (Invitrogen, CA, USA). Then the PCR amplification of the hypervariable regions V3 - V4 of the 16S rRNA gene was carried out according to the Illumina MiSeq’s protocol and using recommended forward (5′-TCGTCGGCAGCGTCAGATGTGTATAAGAGACAGCCTACGGGNGGCWGCAG-3′) and reverse primers (5′-GTCTCGTGGGCTCGGAGATGTGTATAAGAGACAGGACTACHVGGGTATCTAATCC-3’) [29]. The KAPA HiFi HotStart ReadyMix PCR kit (KK2602) was employed to perform the amplification. The PCR reaction included an initial denaturation step of 95°C for 3 min; 25 amplification cycles (95°C for 30 s, 55°C for 30 s and 72°C for 30 s); and a final extension step of 72°C for 5 min. Then, Illumina sequencing adapters and dual-index barcodes (Nextera XT index kit v2, FC-131-2001) were added to the amplicons. Prior to sequencing, libraries were normalized and merged. To improve the sequencing’s quality, the pool with the indexed amplicons was loaded onto the MiSeq reagent cartridge v3 (MS-102-3003) and spiked with 10% PhiX control. The Illumina MiSeq sequencing system was used to perform the paired-end sequencing (2 × 300 bp). Raw Illumina sequences were analyzed using QIIME 2 [30,p.2]. The quality of the reads was assessed with the Demux plugin, and the sequences were subsequently corrected, trimmed, and clustered into amplicon sequence variants (ASVs; > 99.9% of similarity) via Dada2. The taxonomy of each sequence variant was assigned applying the classify-Sklearn module from the feature-classifier plugin, and SILVA (v. 138) [30] was used as reference database for 16S rRNA assignment. The 16S rRNA gene amplicon data set was deposited in GenBank under the SRA accession numbers SRX16260848-SRX16261049 (BioProject number PRJNA858625).

Alpha diversity (richness of taxa, Shannon’s diversity index (H´), Simpson´s diversity index 1-D) and beta diversity of bacterial communities (Bray-Curtis distance) were analysed using the phyloseq (version 1.40.0) and vegan R (version 2.5–7) packages based on ASVs defined at 99.9% [31]. The non-parametric Wilcoxon signed-rank test in R [stats:wilcox.test(a,b, paired = TRUE or FALSE -depending on the nature of the data-)] was used with a Bonferroni correction for multiplicity adjustment to compare the means of numerical values of alpha diversity estimates and the relative abundances of taxa. Principal coordinate analysis (PCoA) was performed with the R package vegan (version 2.5–7). Boxplots and ordination plots were constructed with the ggplot2 R package (version 3.3.6) and the heatmap with ampvis2 R package (version 2.7.2) [32,p.2]. Rarefaction curves were obtained with iNEXT R package (version 2.0.20) [33]. The non-parametric PERMANOVA test in R [vegan:adonis(); default conditions], with a Bonferroni correction for multiplicity adjustment was used to determine statistical significance of differences in beta diversity of bacterial communities. We used the stats package version as embedded in R version 3.6.3 (www.r-project.org). All the sequences assigned to chloroplasts, mitochondria or eukaryotic species were removed from the ecological analyses.

## Results and discussion

The use of plant-derived antimicrobial compounds such as o-cymen-5-ol in oral care products has gained importance in recent years as an alternative to more conventional antiseptic compounds such as chlorhexidine and triclosan due to the side effects of the latter. Recent studies have shown that the addition of o-cymen-5-ol in combination with zinc salts in toothpastes and mouthwashes are effective in reducing plaque formation, gingival inflammation, bleeding gums and halitosis [25,28,34,35]. Zinc salts are often co-formulated with o-cymen-5-ol because of their antimicrobial activity and their ability to inhibit undesirable enzymatic activities of microbial origin in the mouth [25]. Specifically, zinc salts can reduce the growth of *Streptococcus mutans* and initial plaque formation [36,37]. However, the long-term use of zinc salts, like any other antimicrobial biomolecule, has the potential to promote the emergence of resistant bacteria [38]. O-cymen-5-ol has a more intense anticaries activity than other conventional compounds such as chlorhexidine and chlorine dioxide [39] and has a higher penetration capacity in microbial biofilms compared to triclosan or cetylpyridinium chloride [40]. The direct inhibitory effect of the o-cymen-5-ol/zinc system against oral pathogens such as *Streptococcus mutans*, *Actinomyces viscosus*, *Porphyromonas gingivalis*, *Fusobacterium nucleatum* and *Candida albicans* has already been documented [25]. However, to date, the effect of the o-cymen-5-ol/zinc system on the overall oral microbiome in healthy individuals is unknown. An impairment of the oral microbiome by the recurrent use of a non-selective antimicrobial agent may trigger dysbiosis, thus increasing the likelihood of developing oral pathologies [18].

Following the mouthwash/placebo administration procedure, a total of 200 sequence datasets were obtained. After the bioinformatic quality filtering, all the sequence datasets showed a high number of sequences except for sample PLD080T14. For further analysis of paired data, the paired sample of the same volunteer was removed (PLD080T0). The mean number of sequences per sample was 54,529.5 ± 806.8 (minimum = 28946; maximum = 88451) (Figure S1). The rarefaction curves of all samples were saturated, suggesting that the sequencing depth used was sufficient to detect the full diversity of the oral microbiome (Figure S2).

**Figure 1.**
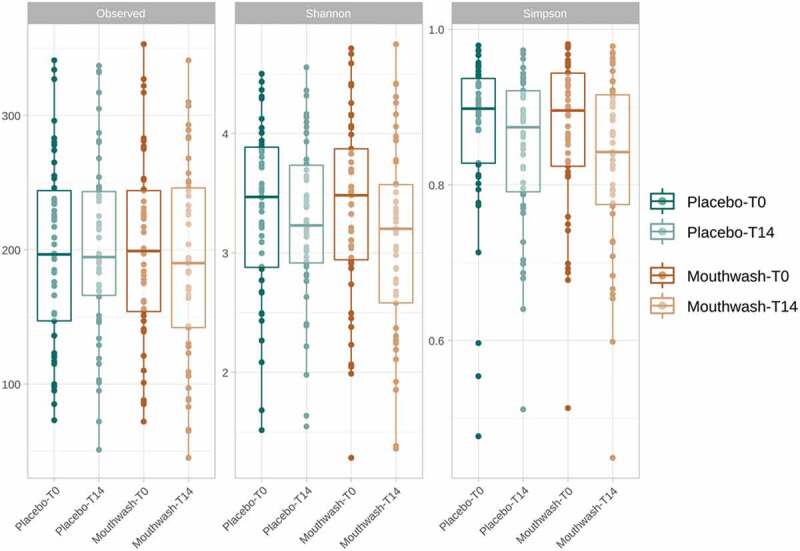
Boxplots of alpha diversity indices (richness observed, Shannon, and Simpson) based on ASVs (99.9% of similarity) of each experimental group (placebo and mouthwash) at each sampling point (T0 and T14 days).

To assess the influence of the mouthwash formulated with o-cymen-5-ol and zinc chloride on the structure of the bacterial community, the alpha and beta diversity were analysed. None of the three alpha diversity indices tested, namely richness, Shannon and Simpson, showed significant differences after 14 days in either the placebo group or the mouthwash group (Wilcoxon signed-rank test for paired data; *p*-value > 0.05) (Figure 1). The mean number of ASVs in the placebo and mouthwash groups was 197.26 ± 9.74 and 200.67 ± 9.68 at T0, and 201.98 ± 9.66 and 190.42 ± 10.31 at T14, respectively. The number of ASVs observed in the two groups was comparable to those observed in other studies of healthy oral microbiomes [41,42]. Although no significant differences were detected, Shannon’s and Simpson’s indices showed a tendency to decrease after 14 days in both groups. This slight decline in diversity indices was reflected in a change in the oral community structure, where a few taxa became dominant throughout the experiment. Since this effect was observed in both groups, the change in the microbiome diversity could be an effect of tooth rinsing. We hypothesise that the decrease in diversity in the mouthwash and placebo groups is because certain volunteers, influenced by the study, brushed their teeth more frequently and/or more intensively than they normally would. Nevertheless, and as mentioned above, the differences found were not significant in any case.

**Figure 2.**
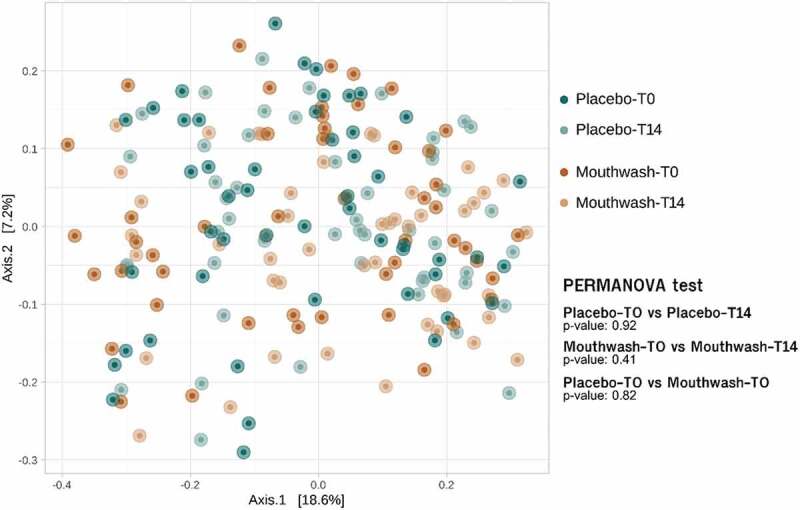
Principal coordinate analysis (PCoA) plot and PERMANOVA tests based on Bray-Curtis distances (ASVs level) of oral microbiome of volunteers enrolled in the mouthwash and placebo groups at T0 and T14 days. Axes represent the two dimensions explaining the greatest proportion of variances in the communities for each analysis.

At the beta diversity level, neither the use of the mouthwash nor the placebo modified the structure of the native oral microbiome of volunteers throughout the study (Figure 2). This result was inferred from the PCoA plot and the PERMANOVA statistic (p-value > 0.05 in both treatment groups throughout the course of treatment). Similar results were obtained when beta diversity was analysed at the bacterial genus level (Fig. S3). When comparing the effect of the mouthwash formulated with o-cymen-5-ol and zinc chloride and placebo by age, range and sex, no significant differences were observed (Fig. S4). Therefore, the oral microbiome was not biased by either the age or the sex of the volunteers.

Previous studies have demonstrated that other antimicrobial compounds typically used in mouthwashes and toothpastes such as chlorhexidine can trigger changes in the structure of the oral microbiome [18,43]. Specifically, these antimicrobial agents reduce the richness and diversity of native species in the mouth because of their broad spectrum of action. Therefore, the mix o-cymen-5-ol/zinc salt does not have the side effects that other antimicrobial agents have such as toxicity and the potential for dysbiosis in the oral microbiome.

**Figure 3.**
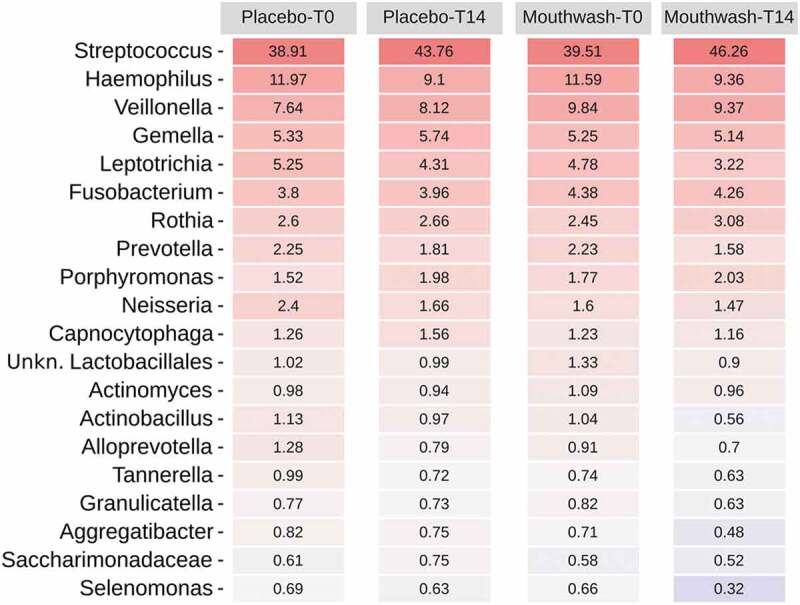
Heatmap of the relative abundances of bacterial genera in both treatment groups at T0 and T14 days.

**Figure 4.**
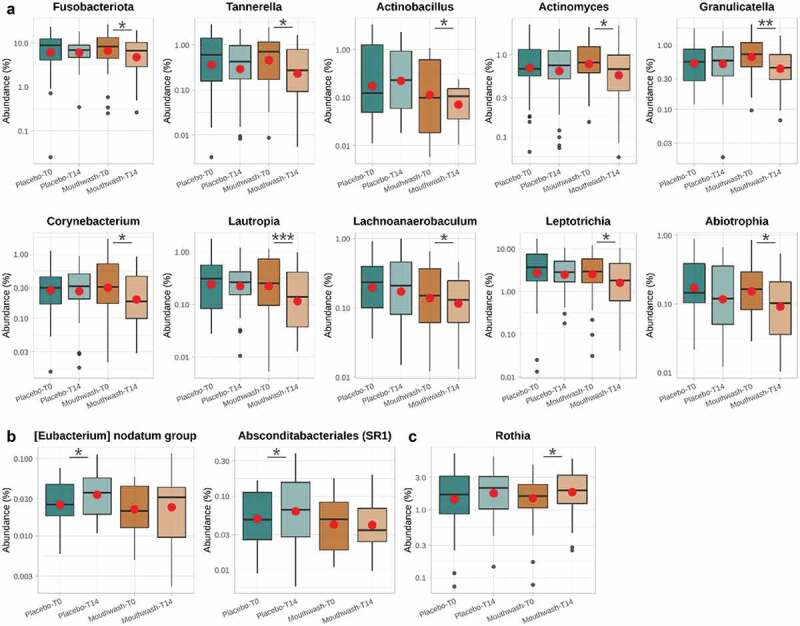
Boxplots of the bacterial genera whose relative abundance (%) showed significant differences throughout the treatment in either of the two groups assessed (Wilcoxon signed-rank test for paired data; *p* < 0.05). *, *p*-value < 0.05; **, *p*-value < 0.01; *** *p*-value < 0.001. Bacteria have been classified according to the dynamics of their relative abundance after treatment. A, bacteria that display a decrease in relative abundance in the mouthwash-treated group; B, bacteria that display an increase in relative abundance in the placebo-treated group; C, bacteria that display an increase in abundance in the group treated with the mouthwash.

The subgingival microbiome of the volunteers at T0 comprised 12 bacterial phyla, the most abundant being *Firmicutes* followed by *Proteobacteria*, *Fusobacteriota*, *Bacteroidota* and *Actinobacteriota* (Fig. S5). This taxonomic structure is comparable to other healthy oral microbiomes previously reported [44–47]. Only one of these 12 phyla, *Fusobacteriota*, showed significant differences after 14 days, being less present in the mouthwash group after treatment (Figure 3) (Wilcoxon signed-rank test for paired data; p-value < 0.05). This phylum has been found to be increased in patients with periodontitis [48], and includes some potential oral pathogen genera such as *Fusobacterium* and *Leptotrichia*. At the genus level, the most abundant taxa were *Streptococcus*, followed by *Haemophilus*, *Veillonella*, *Gemella*, *Leptotrichia* and *Fusobacterium* (Figure 3). Some bacterial genera showed different relative abundances after treatment (Wilcoxon test; p-value < 0.05; Table S3), with these differences being treatment-specific. The relative abundances of some bacteria related to oral pathologies and halitosis (i.e. *Tannerella*, *Actinomyces*, *Granulicatella*, *Abiotrophia, Lautropia* and *Lachnoanaerobaculum*) decreased in the mouthwash-treated group, in contrast to the placebo group (Figure 4a). Similarly, other oral pathogenic bacteria such as *Eubacterium* (*nodatum* group) *and Absconditabacteriales* (SR1) increased their relative abundance in the placebo group throughout the course of treatment (Figure 4b), while remaining constant in the mouthwash group. Therefore, our results demonstrated that the mouthwash formulated with the mix o-cymen-5-ol/zinc salt can control the overgrowth of some pathobiont bacteria [49] and the colonization of pathogenic microorganisms involved in oral and systemic diseases [1]. Other oral pathogens, i.e. *Treponema* or *Porphyromonas*, did not seem to be significantly inhibited by the use of the mouthwash. A decrease in the relative abundance of the genera *Aggregatibacter*, *Neisseria* and *Olsenella* was reported in the placebo group, whereas they remained constant in the mouthwash group (Table S3). This finding suggests that the targeted reduction of these genera in the control group may be due to multifactorial ecological interactions between members of the community. Further studies with symptomatic volunteers would be needed to test if the mouthwash also target these bacterial genera associated with gum diseases and tooth decay.

*Tannerella*, and more specifically *Tannerella forsythia*, is a well-known periodontal pathogen that also can lead to severe systemic diseases such as cardiovascular diseases and even oesophageal cancer [50,51]. This bacterium is one of the three constituents, along with *Porphyromonas gingivalis* and *Treponema denticola*, of the so-called ‘red complex’ in severe periodontal infections. Another genus that specifically decreased its relative abundance in the mouthwash-treated group was *Actinomyces*, which is part of the healthy oral microbiome; however, under a disruption of the environmental balance, mucosal tissue integrity or defense system, it can turn into a pathogen, initiating a prolonged chronic inflammatory process that can lead to the creation of a tumor-like mass, tissue destruction, osteolysis and multiple sinus tracts [52,53]. On the other hand, both *Granulicatella* and *Abiotrophia* are pathobionts of the human mouth linked to endocarditis and pulmonary, central nervous system and ocular infections [54]. As for the *Lautropia* genus, a recent study has shown that the species *L. mirabilis* may be the etiological agent of dialysis-associated peritonitis [55]. Additionally, the increase in the abundance of *Lachnoanaerobaculum* genus has also been associated to periodontal diseases [56]. However, *Leptotrichia* and *Corynebacterium* genera, which were also found to be less abundant after the mouthwash treatment, have proved negatively associated with periodontal clinical parameters [56,57].

As detailed above, other taxa such as *Eubacterium* (*nodatum* group) and *Absconditabacteriales* (SR1) increased their relative abundances in the placebo-treated group, remaining constant in the mouthwash-treated group. The outcomes reveal that a notable percentage of volunteers in the placebo group exhibited an increase in their relative abundances in both taxonomic groups (Table S3). Therefore, the mouthwash slowed down the progression of possible pathogens that can progress in the mouth under normal conditions. *Eubacterium*, and specifically *nodatum* group, is a genus widely linked to periodontal infections [58]. As of *Absconditabacteriales* (SR1), a recent study confirmed its greater presence in the saliva of subjects with dental caries [59].

Even more interesting were the dynamics of *Rothia*. This genus increased its relative abundance in the mouthwash-treated group and not in the placebo-treated group (Figure 4c), with this being a beneficial result for oral health. *Rothia* is a nitrate-reducing oral bacteria which can contribute to prevent oral diseases, as well as increase systemic nitric oxide levels that can improve conditions such as hypertension and diabetes [60]. This effect is opposite to the one that CHX has on the oral microorganisms responsible for metabolizing nitrate and hance regulating blood pressure saliva [15,18]. To date, we do not know which compound in the mouthwash is responsible for promoting the growth of *Rothia*. Whether this is a direct consequence of a compound in the mouthwash or whether it is due to an indirect effect of pathobiont inhibition will be investigated in the future.

## Conclusions

In the present study, we have demonstrated that a mouthwash formulated with the mix o-cymen-5-ol/zinc salts can selectively inhibit or slow down the growth of some common oral pathobionts, including *Prevotella*, *Actinomyces*, *Granulicatella*, *Abiotrophia*, *Lautropia, Lachnoanaerobaculum*, *Eubacterium* (*nodatum* group) and *Absconditabacteriales* (SR1), but without perturbing the native oral microbiome. Although the reduction in the relative abundance of pathobionts is primarily due to the inhibitory effect of the compounds formulated in the mouthwash, it cannot be excluded that the reduction in the abundance of certain taxa is due to a bacterial coaggregation effect. This result gives the o-cymen-5-ol/zinc salts mixture an advantage over traditional antimicrobial agents that are characterised by inducing severe drastic changes in the oral microbiome. On the other hand, the tested mouthwash promotes the growth a nitrate oxidizing microorganism, namely *Rothia*, which is beneficial for regulating blood pressure. Therefore, our study paves the way for the implementation of mouthwashes based on phytochemicals, or their derivatives such as o-cymen-5-ol, as antimicrobial agents able to tackle specific oral pathogens without altering the native microbiome. A future research to evaluate the effect of the mix o-cymen-5-ol/zinc salts on patients with periodontal disease is planned, in order to validate the inhibition of pathobiont when volunteers already have a pathology. To our knowledge, this is the first study addressed to evaluate the effect of a mouthwash formulated with o-cymen-5-ol and zinc chloride on the oral microbiome in healthy individuals.

## Supplementary Material

Supplemental Material

Supplemental Material

Supplemental Material

## Data Availability

All raw sequencing data acquired in this study have been deposited to the National Center for Biotechnology Information Sequence Read Archive (NCBI/SRA) (www.ncbi.nlm.ni.gov/sra) under BioProjectID PRJNA858625.
