# Supplementary material for: A mouthwash formulated with o-cymen-5-ol and zinc chloride specifically targets potential pathogens without impairing the native oral microbiome in healthy individuals
Source: J Oral Microbiol. 2023 Mar 3;15(1):2185962. doi: 10.1080/20002297.2023.2185962 (PMC9987754; doi:10.1080/20002297.2023.2185962)
Supplement: Supplemental Material [file ZJOM_A_2185962_SM2892.zip › Supplementary files/Table_S3.docx]

| **Genus** | **Placebo T0 relative abundance average** | **Placebo T14 relative abundance average** | **Placebo Wilcoxon pvalue** | **Placebo Significance** | **Placebo trends Up Down Equal** | **Mouthwash T0 relative abundance average** | **Mouthwash T14 relative abundance average** | **Mouthwash Wilcoxon pvalue** | **Mouthwash Significance** | **Mouthwash trends Up Down Equal** |
| --- | --- | --- | --- | --- | --- | --- | --- | --- | --- | --- |
| [Eubacterium] brachy group | 0.018 ± 0.004 | 0.042 ± 0.009 | 0.028 | * | 47.5%-30.0%-22.5% | 0.037 ± 0.007 | 0.061 ± 0.012 | 0.019 | * | 50.0%-31.0%-19.0% |
| [Eubacterium] nodatum group | 0.012 ± 0.003 | 0.023 ± 0.005 | 0.025 | * | 42.5%-20.0%-37.5% | 0.012 ± 0.003 | 0.016 ± 0.005 | 0.927 | NS | 23.1%-35.9%-41.0% |
| [Eubacterium] saphenum group | 0.005 ± 0.002 | 0.012 ± 0.004 | 0.071 | NS | 20.5%-10.3%-69.2% | 0.004 ± 0.001 | 0.007 ± 0.003 | 0.311 | NS | 20.0%-12.5%-67.5% |
| [Eubacterium] yurii group | 0.005 ± 0.002 | 0.01 ± 0.003 | 0.109 | NS | 25.6%-10.3%-64.1% | 0.012 ± 0.003 | 0.014 ± 0.003 | 0.62 | NS | 31.8%-27.3%-40.9% |
| Abiotrophia | 0.144 ± 0.035 | 0.147 ± 0.029 | 0.57 | NS | 45.2%-28.6%-26.2% | 0.14 ± 0.032 | 0.075 ± 0.021 | 0.02 | * | 12.8%-51.3%-35.9% |
| Absconditabacteriales (SR1) | 0.043 ± 0.008 | 0.073 ± 0.015 | 0.038 | * | 51.2%-27.9%-20.9% | 0.039 ± 0.008 | 0.039 ± 0.008 | 0.75 | NS | 45.9%-29.7%-24.3% |
| Actinobacillus | 0.255 ± 0.107 | 0.231 ± 0.082 | 0.879 | NS | 20.0%-25.0%-55.0% | 0.203 ± 0.054 | 0.048 ± 0.011 | 0.04 | * | 26.8%-48.8%-24.4% |
| Actinomyces | 0.866 ± 0.083 | 0.821 ± 0.074 | 0.512 | NS | 44.7%-55.3%-0.0% | 0.888 ± 0.073 | 0.723 ± 0.075 | 0.029 | * | 36.6%-63.4%-0.0% |
| Aggregatibacter | 0.765 ± 0.106 | 0.451 ± 0.059 | 0.004 | ** | 29.5%-63.6%-6.8% | 0.489 ± 0.064 | 0.391 ± 0.062 | 0.183 | NS | 42.9%-52.4%-4.8% |
| Alloprevotella | 0.447 ± 0.078 | 0.314 ± 0.047 | 0.395 | NS | 43.6%-56.4%-0.0% | 0.35 ± 0.068 | 0.325 ± 0.059 | 0.964 | NS | 46.3%-53.7%-0.0% |
| Anaeroglobus | 0.005 ± 0.002 | 0.003 ± 0.001 | 0.209 | NS | 10.5%-21.1%-68.4% | 0.01 ± 0.003 | 0.007 ± 0.002 | 0.278 | NS | 12.8%-28.2%-59.0% |
| Atopobium | 0.032 ± 0.007 | 0.04 ± 0.008 | 0.531 | NS | 37.2%-34.9%-27.9% | 0.026 ± 0.005 | 0.048 ± 0.011 | 0.069 | NS | 50.0%-28.9%-21.1% |
| Bergeyella | 0.34 ± 0.037 | 0.363 ± 0.036 | 0.514 | NS | 52.3%-45.5%-2.3% | 0.303 ± 0.032 | 0.225 ± 0.03 | 0.093 | NS | 39.5%-60.5%-0.0% |
| Campylobacter | 0.317 ± 0.041 | 0.328 ± 0.046 | 0.738 | NS | 53.8%-46.2%-0.0% | 0.362 ± 0.054 | 0.354 ± 0.048 | 0.923 | NS | 44.2%-55.8%-0.0% |
| Candidatus Saccharimonas | 0.022 ± 0.004 | 0.033 ± 0.007 | 0.264 | NS | 45.0%-32.5%-22.5% | 0.024 ± 0.005 | 0.026 ± 0.004 | 0.804 | NS | 42.9%-38.1%-19.0% |
| Capnocytophaga | 1.017 ± 0.131 | 1.259 ± 0.187 | 0.454 | NS | 58.7%-41.3%-0.0% | 0.92 ± 0.111 | 1.015 ± 0.132 | 0.666 | NS | 47.8%-52.2%-0.0% |
| Cardiobacterium | 0.288 ± 0.048 | 0.214 ± 0.04 | 0.637 | NS | 42.5%-52.5%-5.0% | 0.204 ± 0.035 | 0.154 ± 0.029 | 0.204 | NS | 38.5%-59.0%-2.6% |
| Catonella | 0.014 ± 0.003 | 0.02 ± 0.004 | 0.264 | NS | 40.9%-29.5%-29.5% | 0.014 ± 0.004 | 0.018 ± 0.004 | 0.605 | NS | 35.0%-22.5%-42.5% |
| Centipeda | 0.024 ± 0.006 | 0.019 ± 0.006 | 0.277 | NS | 26.3%-31.6%-42.1% | 0.012 ± 0.004 | 0.019 ± 0.005 | 0.248 | NS | 28.2%-17.9%-53.8% |
| Clostridia UCG-014 | 0.069 ± 0.014 | 0.045 ± 0.007 | 0.209 | NS | 43.9%-43.9%-12.2% | 0.097 ± 0.018 | 0.057 ± 0.01 | 0.074 | NS | 34.9%-55.8%-9.3% |
| Corynebacterium | 0.351 ± 0.042 | 0.304 ± 0.036 | 0.639 | NS | 45.5%-50.0%-4.5% | 0.442 ± 0.062 | 0.261 ± 0.038 | 0.014 | * | 37.8%-62.2%-0.0% |
| Dialister | 0.143 ± 0.028 | 0.089 ± 0.02 | 0.115 | NS | 35.7%-61.9%-2.4% | 0.112 ± 0.019 | 0.084 ± 0.014 | 0.106 | NS | 29.3%-58.5%-12.2% |
| Eikenella | 0.037 ± 0.007 | 0.031 ± 0.007 | 0.357 | NS | 34.1%-41.5%-24.4% | 0.047 ± 0.009 | 0.067 ± 0.011 | 0.126 | NS | 46.8%-34.0%-19.1% |
| F0058 | 0.118 ± 0.027 | 0.133 ± 0.027 | 0.756 | NS | 45.0%-42.5%-12.5% | 0.068 ± 0.013 | 0.065 ± 0.012 | 0.985 | NS | 36.8%-47.4%-15.8% |
| F0332 | 0.07 ± 0.016 | 0.06 ± 0.011 | 0.719 | NS | 42.5%-32.5%-25.0% | 0.115 ± 0.022 | 0.078 ± 0.016 | 0.213 | NS | 37.5%-50.0%-12.5% |
| Filifactor | 0.034 ± 0.009 | 0.039 ± 0.009 | 0.412 | NS | 35.0%-27.5%-37.5% | 0.04 ± 0.01 | 0.056 ± 0.014 | 0.098 | NS | 45.2%-26.2%-28.6% |
| Fretibacterium | 0.014 ± 0.004 | 0.005 ± 0.001 | 0.131 | NS | 25.6%-28.2%-46.2% | 0.008 ± 0.002 | 0.013 ± 0.004 | 0.266 | NS | 25.6%-17.9%-56.4% |
| Fusobacterium | 3.5 ± 0.341 | 3.653 ± 0.312 | 0.664 | NS | 51.1%-48.9%-0.0% | 3.814 ± 0.388 | 3.271 ± 0.382 | 0.093 | NS | 40.9%-59.1%-0.0% |
| Gemella | 4.765 ± 0.531 | 5.48 ± 0.552 | 0.282 | NS | 56.2%-43.8%-0.0% | 5.271 ± 0.572 | 4.657 ± 0.374 | 0.559 | NS | 54.3%-45.7%-0.0% |
| Granulicatella | 0.662 ± 0.065 | 0.663 ± 0.06 | 0.782 | NS | 54.2%-45.8%-0.0% | 0.812 ± 0.072 | 0.529 ± 0.048 | 0.002 | ** | 34.0%-66.0%-0.0% |
| Haemophilus | 10.737 ± 0.947 | 8.368 ± 0.863 | 0.069 | NS | 38.3%-61.7%-0.0% | 9.499 ± 0.78 | 9.243 ± 0.985 | 0.735 | NS | 47.7%-52.3%-0.0% |
| JGI 0000069-P22 | 0.007 ± 0.002 | 0.014 ± 0.003 | 0.067 | NS | 36.6%-22.0%-41.5% | 0.005 ± 0.002 | 0.005 ± 0.002 | 0.975 | NS | 21.6%-16.2%-62.2% |
| Johnsonella | 0.051 ± 0.011 | 0.051 ± 0.009 | 0.66 | NS | 47.6%-38.1%-14.3% | 0.073 ± 0.016 | 0.06 ± 0.012 | 0.604 | NS | 41.5%-46.3%-12.2% |
| Kingella | 0.167 ± 0.025 | 0.14 ± 0.02 | 0.429 | NS | 39.0%-53.7%-7.3% | 0.175 ± 0.022 | 0.216 ± 0.028 | 0.396 | NS | 54.8%-42.9%-2.4% |
| Lachnoanaerobaculum | 0.257 ± 0.036 | 0.273 ± 0.039 | 0.705 | NS | 52.2%-45.7%-2.2% | 0.228 ± 0.031 | 0.146 ± 0.019 | 0.032 | * | 36.4%-63.6%-0.0% |
| Lautropia | 0.407 ± 0.077 | 0.298 ± 0.045 | 0.722 | NS | 52.5%-42.5%-5.0% | 0.376 ± 0.054 | 0.216 ± 0.039 | 0.001 | *** | 24.4%-68.9%-6.7% |
| Lentimicrobium | 0.026 ± 0.007 | 0.025 ± 0.006 | 0.809 | NS | 37.5%-27.5%-35.0% | 0.014 ± 0.004 | 0.016 ± 0.004 | 0.783 | NS | 30.0%-25.0%-45.0% |
| Leptotrichia | 4.817 ± 0.598 | 3.504 ± 0.421 | 0.071 | NS | 34.8%-65.2%-0.0% | 3.916 ± 0.457 | 2.912 ± 0.416 | 0.035 | * | 39.1%-60.9%-0.0% |
| Mycoplasma | 0.006 ± 0.002 | 0.011 ± 0.003 | 0.126 | NS | 30.0%-17.5%-52.5% | 0.009 ± 0.002 | 0.006 ± 0.002 | 0.189 | NS | 23.1%-33.3%-43.6% |
| Neisseria | 1.968 ± 0.267 | 1.296 ± 0.183 | 0.013 | * | 31.9%-66.0%-2.1% | 1.102 ± 0.155 | 0.821 ± 0.099 | 0.197 | NS | 45.0%-55.0%-0.0% |
| Olsenella | 0.033 ± 0.008 | 0.007 ± 0.002 | 0.004 | ** | 20.0%-40.0%-40.0% | 0.024 ± 0.005 | 0.018 ± 0.003 | 0.358 | NS | 29.3%-41.5%-29.3% |
| Oribacterium | 0.044 ± 0.009 | 0.06 ± 0.011 | 0.397 | NS | 45.0%-40.0%-15.0% | 0.049 ± 0.008 | 0.061 ± 0.011 | 0.379 | NS | 42.5%-42.5%-15.0% |
| Parvimonas | 0.1 ± 0.02 | 0.095 ± 0.015 | 0.944 | NS | 46.5%-44.2%-9.3% | 0.087 ± 0.018 | 0.127 ± 0.025 | 0.252 | NS | 50.0%-37.5%-12.5% |
| Peptococcus | 0.019 ± 0.004 | 0.024 ± 0.004 | 0.278 | NS | 43.9%-34.1%-22.0% | 0.024 ± 0.005 | 0.023 ± 0.005 | 0.766 | NS | 31.8%-36.4%-31.8% |
| Peptostreptococcus | 0.051 ± 0.013 | 0.084 ± 0.015 | 0.081 | NS | 51.2%-36.6%-12.2% | 0.052 ± 0.012 | 0.057 ± 0.012 | 0.837 | NS | 41.0%-43.6%-15.4% |
| Porphyromonas | 1.167 ± 0.13 | 1.447 ± 0.167 | 0.273 | NS | 56.8%-43.2%-0.0% | 1.439 ± 0.151 | 1.706 ± 0.223 | 0.753 | NS | 43.2%-56.8%-0.0% |
| Prevotella | 1.706 ± 0.192 | 1.564 ± 0.159 | 0.857 | NS | 47.8%-52.2%-0.0% | 1.967 ± 0.27 | 1.33 ± 0.155 | 0.08 | NS | 41.3%-58.7%-0.0% |
| Pseudopropionibacterium | 0.004 ± 0.001 | 0.004 ± 0.001 | 0.936 | NS | 25.6%-23.1%-51.3% | 0.006 ± 0.002 | 0.007 ± 0.002 | 0.717 | NS | 22.5%-17.5%-60.0% |
| Rothia | 2.086 ± 0.235 | 2.215 ± 0.204 | 0.569 | NS | 51.1%-48.9%-0.0% | 1.878 ± 0.18 | 2.289 ± 0.217 | 0.045 | * | 59.5%-40.5%-0.0% |
| Saccharimonadaceae | 0.35 ± 0.061 | 0.312 ± 0.053 | 0.936 | NS | 51.2%-46.3%-2.4% | 0.352 ± 0.056 | 0.485 ± 0.078 | 0.209 | NS | 53.3%-42.2%-4.4% |
| Saccharimonadales | 0.079 ± 0.019 | 0.115 ± 0.027 | 0.549 | NS | 38.1%-40.5%-21.4% | 0.126 ± 0.027 | 0.099 ± 0.017 | 0.589 | NS | 41.5%-43.9%-14.6% |
| Selenomonas | 0.37 ± 0.062 | 0.227 ± 0.044 | 0.098 | NS | 41.5%-56.1%-2.4% | 0.233 ± 0.037 | 0.206 ± 0.035 | 0.536 | NS | 47.5%-52.5%-0.0% |
| Solobacterium | 0.03 ± 0.006 | 0.023 ± 0.004 | 0.41 | NS | 39.1%-45.7%-15.2% | 0.021 ± 0.003 | 0.029 ± 0.006 | 0.406 | NS | 44.2%-32.6%-23.3% |
| Stomatobaculum | 0.059 ± 0.013 | 0.044 ± 0.009 | 0.638 | NS | 36.6%-39.0%-24.4% | 0.061 ± 0.01 | 0.042 ± 0.007 | 0.102 | NS | 31.8%-50.0%-18.2% |
| Streptococcus | 37.277 ± 1.345 | 43.315 ± 1.633 | 0.009 | ** | 67.4%-32.6%-0.0% | 38.447 ± 1.761 | 46.346 ± 1.957 | 0 | *** | 75.0%-25.0%-0.0% |
| Tannerella | 0.73 ± 0.114 | 0.574 ± 0.09 | 0.083 | NS | 39.1%-58.7%-2.2% | 0.686 ± 0.092 | 0.43 ± 0.069 | 0.031 | * | 38.6%-59.1%-2.3% |
| TM7x | 0.114 ± 0.021 | 0.101 ± 0.016 | 0.958 | NS | 53.8%-41.0%-5.1% | 0.181 ± 0.03 | 0.159 ± 0.025 | 0.308 | NS | 43.2%-52.3%-4.5% |
| Treponema | 0.355 ± 0.075 | 0.234 ± 0.041 | 0.957 | NS | 51.2%-41.9%-7.0% | 0.228 ± 0.045 | 0.174 ± 0.032 | 0.226 | NS | 41.5%-46.3%-12.2% |
| Veillonella | 7.03 ± 0.572 | 7.201 ± 0.638 | 0.674 | NS | 56.5%-43.5%-0.0% | 9.835 ± 1.017 | 9.37 ± 0.89 | 0.917 | NS | 55.1%-44.9%-0.0% |
