# Supplementary material for: A mouthwash formulated with o-cymen-5-ol and zinc chloride specifically targets potential pathogens without impairing the native oral microbiome in healthy individuals
Source: J Oral Microbiol. 2023 Mar 3;15(1):2185962. doi: 10.1080/20002297.2023.2185962 (PMC9987754; doi:10.1080/20002297.2023.2185962)
Supplement: Supplemental Material [file ZJOM_A_2185962_SM6415.docx]

| **aGenus** | **Placebo T0 relative abundance average** | **Placebo T14 relative abundance average** | **Placebo Wilcoxon pvalue** | **Placebo Significance** | **Placebo trends Up Down Equal** | **Mouthwash T0 relative abundance average** | **Mouthwash T14 relative abundance average** | **Mouthwash Wilcoxon pvalue** | **Mouthwash Significance** | **Mouthwash trends Up Down Equal** |
| --- | --- | --- | --- | --- | --- | --- | --- | --- | --- | --- |
| [Eubacterium] brachy group | 0.018 ± 0.004 | 0.042 ± 0.009 | 0.028 | * | 47.5%-30.0%-22.5% | 0.037 ± 0.007 | 0.061 ± 0.012 | 0.019 | * | 50.0%-31.0%-19.0% |
| [Eubacterium] nodatum group | 0.012 ± 0.003 | 0.023 ± 0.005 | 0.025 | * | 42.5%-20.0%-37.5% | 0.012 ± 0.003 | 0.016 ± 0.005 | 0.927 | NS | 23.1%-35.9%-41.0% |
| [Eubacterium] saphenum group | 0.005 ± 0.002 | 0.012 ± 0.004 | 0.071 | NS | 20.5%-10.3%-69.2% | 0.004 ± 0.001 | 0.007 ± 0.003 | 0.311 | NS | 20.0%-12.5%-67.5% |
| [Eubacterium] yurii group | 0.005 ± 0.002 | 0.01 ± 0.003 | 0.109 | NS | 25.6%-10.3%-64.1% | 0.012 ± 0.003 | 0.014 ± 0.003 | 0.62 | NS | 31.8%-27.3%-40.9% |
| Abiotrophia | 0.144 ± 0.035 | 0.147 ± 0.029 | 0.57 | NS | 45.2%-28.6%-26.2% | 0.14 ± 0.032 | 0.075 ± 0.021 | 0.02 | * | 12.8%-51.3%-35.9% |
| Absconditabacteriales (SR1) | 0.043 ± 0.008 | 0.073 ± 0.015 | 0.038 | * | 51.2%-27.9%-20.9% | 0.039 ± 0.008 | 0.039 ± 0.008 | 0.75 | NS | 45.9%-29.7%-24.3% |
| Actinobacillus | 0.255 ± 0.107 | 0.231 ± 0.082 | 0.879 | NS | 20.0%-25.0%-55.0% | 0.203 ± 0.054 | 0.048 ± 0.011 | 0.04 | * | 26.8%-48.8%-24.4% |
| Actinomyces | 0.866 ± 0.083 | 0.821 ± 0.074 | 0.512 | NS | 44.7%-55.3%-0.0% | 0.888 ± 0.073 | 0.723 ± 0.075 | 0.029 | * | 36.6%-63.4%-0.0% |
| Aggregatibacter | 0.765 ± 0.106 | 0.451 ± 0.059 | 0.004 | ** | 29.5%-63.6%-6.8% | 0.489 ± 0.064 | 0.391 ± 0.062 | 0.183 | NS | 42.9%-52.4%-4.8% |
| Alloprevotella | 0.447 ± 0.078 | 0.314 ± 0.047 | 0.395 | NS | 43.6%-56.4%-0.0% | 0.35 ± 0.068 | 0.325 ± 0.059 | 0.964 | NS | 46.3%-53.7%-0.0% |
| Anaeroglobus | 0.005 ± 0.002 | 0.003 ± 0.001 | 0.209 | NS | 10.5%-21.1%-68.4% | 0.01 ± 0.003 | 0.007 ± 0.002 | 0.278 | NS | 12.8%-28.2%-59.0% |
| Atopobium | 0.032 ± 0.007 | 0.04 ± 0.008 | 0.531 | NS | 37.2%-34.9%-27.9% | 0.026 ± 0.005 | 0.048 ± 0.011 | 0.069 | NS | 50.0%-28.9%-21.1% |
| Bergeyella | 0.34 ± 0.037 | 0.363 ± 0.036 | 0.514 | NS | 52.3%-45.5%-2.3% | 0.303 ± 0.032 | 0.225 ± 0.03 | 0.093 | NS | 39.5%-60.5%-0.0% |
| Campylobacter | 0.317 ± 0.041 | 0.328 ± 0.046 | 0.738 | NS | 53.8%-46.2%-0.0% | 0.362 ± 0.054 | 0.354 ± 0.048 | 0.923 | NS | 44.2%-55.8%-0.0% |
| Candidatus Saccharimonas | 0.022 ± 0.004 | 0.033 ± 0.007 | 0.264 | NS | 45.0%-32.5%-22.5% | 0.024 ± 0.005 | 0.026 ± 0.004 | 0.804 | NS | 42.9%-38.1%-19.0% |
| Capnocytophaga | 1.017 ± 0.131 | 1.259 ± 0.187 | 0.454 | NS | 58.7%-41.3%-0.0% | 0.92 ± 0.111 | 1.015 ± 0.132 | 0.666 | NS | 47.8%-52.2%-0.0% |
| Cardiobacterium | 0.288 ± 0.048 | 0.214 ± 0.04 | 0.637 | NS | 42.5%-52.5%-5.0% | 0.204 ± 0.035 | 0.154 ± 0.029 | 0.204 | NS | 38.5%-59.0%-2.6% |
| Catonella | 0.014 ± 0.003 | 0.02 ± 0.004 | 0.264 | NS | 40.9%-29.5%-29.5% | 0.014 ± 0.004 | 0.018 ± 0.004 | 0.605 | NS | 35.0%-22.5%-42.5% |
| Centipeda | 0.024 ± 0.006 | 0.019 ± 0.006 | 0.277 | NS | 26.3%-31.6%-42.1% | 0.012 ± 0.004 | 0.019 ± 0.005 | 0.248 | NS | 28.2%-17.9%-53.8% |
| Clostridia UCG-014 | 0.069 ± 0.014 | 0.045 ± 0.007 | 0.209 | NS | 43.9%-43.9%-12.2% | 0.097 ± 0.018 | 0.057 ± 0.01 | 0.074 | NS | 34.9%-55.8%-9.3% |
| Corynebacterium | 0.351 ± 0.042 | 0.304 ± 0.036 | 0.639 | NS | 45.5%-50.0%-4.5% | 0.442 ± 0.062 | 0.261 ± 0.038 | 0.014 | * | 37.8%-62.2%-0.0% |
| Dialister | 0.143 ± 0.028 | 0.089 ± 0.02 | 0.115 | NS | 35.7%-61.9%-2.4% | 0.112 ± 0.019 | 0.084 ± 0.014 | 0.106 | NS | 29.3%-58.5%-12.2% |
| Eikenella | 0.037 ± 0.007 | 0.031 ± 0.007 | 0.357 | NS | 34.1%-41.5%-24.4% | 0.047 ± 0.009 | 0.067 ± 0.011 | 0.126 | NS | 46.8%-34.0%-19.1% |
| F0058 | 0.118 ± 0.027 | 0.133 ± 0.027 | 0.756 | NS | 45.0%-42.5%-12.5% | 0.068 ± 0.013 | 0.065 ± 0.012 | 0.985 | NS | 36.8%-47.4%-15.8% |
| F0332 | 0.07 ± 0.016 | 0.06 ± 0.011 | 0.719 | NS | 42.5%-32.5%-25.0% | 0.115 ± 0.022 | 0.078 ± 0.016 | 0.213 | NS | 37.5%-50.0%-12.5% |
| Filifactor | 0.034 ± 0.009 | 0.039 ± 0.009 | 0.412 | NS | 35.0%-27.5%-37.5% | 0.04 ± 0.01 | 0.056 ± 0.014 | 0.098 | NS | 45.2%-26.2%-28.6% |
| Fretibacterium | 0.014 ± 0.004 | 0.005 ± 0.001 | 0.131 | NS | 25.6%-28.2%-46.2% | 0.008 ± 0.002 | 0.013 ± 0.004 | 0.266 | NS | 25.6%-17.9%-56.4% |
| Fusobacterium | 3.5 ± 0.341 | 3.653 ± 0.312 | 0.664 | NS | 51.1%-48.9%-0.0% | 3.814 ± 0.388 | 3.271 ± 0.382 | 0.093 | NS | 40.9%-59.1%-0.0% |
| Gemella | 4.765 ± 0.531 | 5.48 ± 0.552 | 0.282 | NS | 56.2%-43.8%-0.0% | 5.271 ± 0.572 | 4.657 ± 0.374 | 0.559 | NS | 54.3%-45.7%-0.0% |
| Granulicatella | 0.662 ± 0.065 | 0.663 ± 0.06 | 0.782 | NS | 54.2%-45.8%-0.0% | 0.812 ± 0.072 | 0.529 ± 0.048 | 0.002 | ** | 34.0%-66.0%-0.0% |
| Haemophilus | 10.737 ± 0.947 | 8.368 ± 0.863 | 0.069 | NS | 38.3%-61.7%-0.0% | 9.499 ± 0.78 | 9.243 ± 0.985 | 0.735 | NS | 47.7%-52.3%-0.0% |
| JGI 0000069-P22 | 0.007 ± 0.002 | 0.014 ± 0.003 | 0.067 | NS | 36.6%-22.0%-41.5% | 0.005 ± 0.002 | 0.005 ± 0.002 | 0.975 | NS | 21.6%-16.2%-62.2% |
| Johnsonella | 0.051 ± 0.011 | 0.051 ± 0.009 | 0.66 | NS | 47.6%-38.1%-14.3% | 0.073 ± 0.016 | 0.06 ± 0.012 | 0.604 | NS | 41.5%-46.3%-12.2% |
| Kingella | 0.167 ± 0.025 | 0.14 ± 0.02 | 0.429 | NS | 39.0%-53.7%-7.3% | 0.175 ± 0.022 | 0.216 ± 0.028 | 0.396 | NS | 54.8%-42.9%-2.4% |
| Lachnoanaerobaculum | 0.257 ± 0.036 | 0.273 ± 0.039 | 0.705 | NS | 52.2%-45.7%-2.2% | 0.228 ± 0.031 | 0.146 ± 0.019 | 0.032 | * | 36.4%-63.6%-0.0% |
| Lautropia | 0.407 ± 0.077 | 0.298 ± 0.045 | 0.722 | NS | 52.5%-42.5%-5.0% | 0.376 ± 0.054 | 0.216 ± 0.039 | 0.001 | *** | 24.4%-68.9%-6.7% |
| Lentimicrobium | 0.026 ± 0.007 | 0.025 ± 0.006 | 0.809 | NS | 37.5%-27.5%-35.0% | 0.014 ± 0.004 | 0.016 ± 0.004 | 0.783 | NS | 30.0%-25.0%-45.0% |
| Leptotrichia | 4.817 ± 0.598 | 3.504 ± 0.421 | 0.071 | NS | 34.8%-65.2%-0.0% | 3.916 ± 0.457 | 2.912 ± 0.416 | 0.035 | * | 39.1%-60.9%-0.0% |
| Mycoplasma | 0.006 ± 0.002 | 0.011 ± 0.003 | 0.126 | NS | 30.0%-17.5%-52.5% | 0.009 ± 0.002 | 0.006 ± 0.002 | 0.189 | NS | 23.1%-33.3%-43.6% |
| Neisseria | 1.968 ± 0.267 | 1.296 ± 0.183 | 0.013 | * | 31.9%-66.0%-2.1% | 1.102 ± 0.155 | 0.821 ± 0.099 | 0.197 | NS | 45.0%-55.0%-0.0% |
| Olsenella | 0.033 ± 0.008 | 0.007 ± 0.002 | 0.004 | ** | 20.0%-40.0%-40.0% | 0.024 ± 0.005 | 0.018 ± 0.003 | 0.358 | NS | 29.3%-41.5%-29.3% |
| Oribacterium | 0.044 ± 0.009 | 0.06 ± 0.011 | 0.397 | NS | 45.0%-40.0%-15.0% | 0.049 ± 0.008 | 0.061 ± 0.011 | 0.379 | NS | 42.5%-42.5%-15.0% |
| Parvimonas | 0.1 ± 0.02 | 0.095 ± 0.015 | 0.944 | NS | 46.5%-44.2%-9.3% | 0.087 ± 0.018 | 0.127 ± 0.025 | 0.252 | NS | 50.0%-37.5%-12.5% |
| Peptococcus | 0.019 ± 0.004 | 0.024 ± 0.004 | 0.278 | NS | 43.9%-34.1%-22.0% | 0.024 ± 0.005 | 0.023 ± 0.005 | 0.766 | NS | 31.8%-36.4%-31.8% |
| Peptostreptococcus | 0.051 ± 0.013 | 0.084 ± 0.015 | 0.081 | NS | 51.2%-36.6%-12.2% | 0.052 ± 0.012 | 0.057 ± 0.012 | 0.837 | NS | 41.0%-43.6%-15.4% |
| Porphyromonas | 1.167 ± 0.13 | 1.447 ± 0.167 | 0.273 | NS | 56.8%-43.2%-0.0% | 1.439 ± 0.151 | 1.706 ± 0.223 | 0.753 | NS | 43.2%-56.8%-0.0% |
| Prevotella | 1.706 ± 0.192 | 1.564 ± 0.159 | 0.857 | NS | 47.8%-52.2%-0.0% | 1.967 ± 0.27 | 1.33 ± 0.155 | 0.08 | NS | 41.3%-58.7%-0.0% |
| Pseudopropionibacterium | 0.004 ± 0.001 | 0.004 ± 0.001 | 0.936 | NS | 25.6%-23.1%-51.3% | 0.006 ± 0.002 | 0.007 ± 0.002 | 0.717 | NS | 22.5%-17.5%-60.0% |
| Rothia | 2.086 ± 0.235 | 2.215 ± 0.204 | 0.569 | NS | 51.1%-48.9%-0.0% | 1.878 ± 0.18 | 2.289 ± 0.217 | 0.045 | * | 59.5%-40.5%-0.0% |
| Saccharimonadaceae | 0.35 ± 0.061 | 0.312 ± 0.053 | 0.936 | NS | 51.2%-46.3%-2.4% | 0.352 ± 0.056 | 0.485 ± 0.078 | 0.209 | NS | 53.3%-42.2%-4.4% |
| Saccharimonadales | 0.079 ± 0.019 | 0.115 ± 0.027 | 0.549 | NS | 38.1%-40.5%-21.4% | 0.126 ± 0.027 | 0.099 ± 0.017 | 0.589 | NS | 41.5%-43.9%-14.6% |
| Selenomonas | 0.37 ± 0.062 | 0.227 ± 0.044 | 0.098 | NS | 41.5%-56.1%-2.4% | 0.233 ± 0.037 | 0.206 ± 0.035 | 0.536 | NS | 47.5%-52.5%-0.0% |
| Solobacterium | 0.03 ± 0.006 | 0.023 ± 0.004 | 0.41 | NS | 39.1%-45.7%-15.2% | 0.021 ± 0.003 | 0.029 ± 0.006 | 0.406 | NS | 44.2%-32.6%-23.3% |
| Stomatobaculum | 0.059 ± 0.013 | 0.044 ± 0.009 | 0.638 | NS | 36.6%-39.0%-24.4% | 0.061 ± 0.01 | 0.042 ± 0.007 | 0.102 | NS | 31.8%-50.0%-18.2% |
| Streptococcus | 37.277 ± 1.345 | 43.315 ± 1.633 | 0.009 | ** | 67.4%-32.6%-0.0% | 38.447 ± 1.761 | 46.346 ± 1.957 | 0 | *** | 75.0%-25.0%-0.0% |
| Tannerella | 0.73 ± 0.114 | 0.574 ± 0.09 | 0.083 | NS | 39.1%-58.7%-2.2% | 0.686 ± 0.092 | 0.43 ± 0.069 | 0.031 | * | 38.6%-59.1%-2.3% |
| TM7x | 0.114 ± 0.021 | 0.101 ± 0.016 | 0.958 | NS | 53.8%-41.0%-5.1% | 0.181 ± 0.03 | 0.159 ± 0.025 | 0.308 | NS | 43.2%-52.3%-4.5% |
| Treponema | 0.355 ± 0.075 | 0.234 ± 0.041 | 0.957 | NS | 51.2%-41.9%-7.0% | 0.228 ± 0.045 | 0.174 ± 0.032 | 0.226 | NS | 41.5%-46.3%-12.2% |
| Veillonella | 7.03 ± 0.572 | 7.201 ± 0.638 | 0.674 | NS | 56.5%-43.5%-0.0% | 9.835 ± 1.017 | 9.37 ± 0.89 | 0.917 | NS | 55.1%-44.9%-0.0% |

**Table S3.** Bacterial genera showing significant changes in at least one of the two treatments (placebo and mouthwash) at the end of the study, with their respective mean relative abundance and standard error, the p value obtained from the Wilcoxon signed-rank test and the corresponding statistical significance (NS, not significant; *, p value <0.05; **, p value <0.01; *** p value <0.001). In addition, the table shows the trends observed within each group and genus, indicating the percentage of samples that showed an increase, decrease or no change in relative abundance after treatment.
