# Supplementary material for: A mouthwash formulated with o-cymen-5-ol and zinc chloride specifically targets potential pathogens without impairing the native oral microbiome in healthy individuals
Source: J Oral Microbiol. 2023 Mar 3;15(1):2185962. doi: 10.1080/20002297.2023.2185962 (PMC9987754; doi:10.1080/20002297.2023.2185962)
Supplement: Supplementary file 4 [file ZJOM_A_2185962_SM5574.odt]

Supplementary Information

Exclusion criteria for recruiting subjects

- Periodontitis, according to the following criteria: 1. detectable interdental clinical attachment level (CAL) on two or more non-adjacent teeth or 2. buccal CAL greater than or equal to 3 mm with cavities greater than 3 mm detectable on two or more teeth.

- Gingival bleeding (bleeding on probing (BoP) > 25%).

- Presence of orthodontics, implants or visible caries.

- Cancer patients; patients participating in another clinical study; pregnancy or lactation.

- Halitosis, oral manifestations of HIV, dental trauma, cleft lip and palate.

- Antibiotic use during the last 3 months.

- Use of illegal drugs.

- Consumption of more than 5 cigarettes per day or vaping of nicotine-containing substances.

- Allergy or reactivity to cosmetic products of the same category as the one being tested.

Figure S1 Number of high-quality sequences obtained in the 200 oral samples.

Figure S2 Rarefaction curves showing interpolated and extrapolated ASVs richness (continuous and dotted lines, respectively) observed for each oral sample. To simplify the visualisation, the 200 samples have been split into 20 batches.

Escala de tiempo

Descripción generada automáticamente con confianza media

Figure S3 Principal coordinate analysis (PCoA) plot and PERMANOVA tests based on Bray-Curtis distances (genus level) of oral microbiome of volunteers enrolled in the mouthwash and placebo groups at T0 and T14 days. Axes represent the two dimensions explaining the greatest proportion of variances in the communities for each analysis.

Figure S4 Principal coordinate analysis (PCoA) plot and PERMANOVA tests based on Bray-Curtis distances (ASV level) of oral microbiome of volunteers enrolled in the mouthwash and placebo groups at T0 and T14 days, grouped by sex (S4A) and age range (S4B).

Figure S5 Heatmap showing the relative abundance (%) of phyla in both treatment groups at T0 and T14 days. 

Table S1. List of 16S rRNA gene amplicon samples analysed in this study. For each oral sample, the volunteer, age, gender, treatment, and trademark of the toothpaste used during the study are shown. Two measurements, T0 and T14 days, were obtained from each volunteer.

| Volunteer | Age | Sex | Toothpaste | Treatment | Time | SampleID |
| --- | --- | --- | --- | --- | --- | --- |
| PLD001 | 23 | M | Oral B | Placebo | T0 | PLD001t0 |
|  |  |  |  |  | T14 | PLD001t14 |
| PLD003 | 54 | F | Colgate | Placebo | T0 | PLD003t0 |
|  |  |  |  |  | T14 | PLD003t14 |
| PLD005 | 44 | F | Colgate | Mouthwash | T0 | PLD005t0 |
|  |  |  |  |  | T14 | PLD005t14 |
| PLD006 | 47 | F | Colgate | Placebo | T0 | PLD006t0 |
|  |  |  |  |  | T14 | PLD006t14 |
| PLD007 | 42 | F | Colgate | Mouthwash | T0 | PLD007t0 |
|  |  |  |  |  | T14 | PLD007t14 |
| PLD008 | 20 | F | Colgate | Mouthwash | T0 | PLD008t0 |
|  |  |  |  |  | T14 | PLD008t14 |
| PLD009 | 28 | F | Licor del polo | Placebo | T0 | PLD009t0 |
|  |  |  |  |  | T14 | PLD009t14 |
| PLD010 | 25 | M | Colgate | Mouthwash | T0 | PLD010t0 |
|  |  |  |  |  | T14 | PLD010t14 |
| PLD011 | 65 | F | Colgate | Placebo | T0 | PLD011t0 |
|  |  |  |  |  | T14 | PLD011t14 |
| PLD012 | 31 | F | Colgate | Mouthwash | T0 | PLD012t0 |
|  |  |  |  |  | T14 | PLD012t14 |
| PLD013 | 33 | M | Bonté | Placebo | T0 | PLD013t0 |
|  |  |  |  |  | T14 | PLD013t14 |
| PLD014 | 32 | M | Colgate | Placebo | T0 | PLD014t0 |
|  |  |  |  |  | T14 | PLD014t14 |
| PLD015 | 31 | F | Colgate | Mouthwash | T0 | PLD015t0 |
|  |  |  |  |  | T14 | PLD015t14 |
| PLD016 | 41 | F | Colgate | Mouthwash | T0 | PLD016t0 |
|  |  |  |  |  | T14 | PLD016t14 |
| PLD017 | 43 | F | Colgate | Placebo | T0 | PLD017t0 |
|  |  |  |  |  | T14 | PLD017t14 |
| PLD018 | 36 | M | Colgate | Placebo | T0 | PLD018t0 |
|  |  |  |  |  | T14 | PLD018t14 |
| PLD019 | 39 | F | Colgate | Mouthwash | T0 | PLD019t0 |
|  |  |  |  |  | T14 | PLD019t14 |
| PLD020 | 52 | F | Colgate | Mouthwash | T0 | PLD020t0 |
|  |  |  |  |  | T14 | PLD020t14 |
| PLD021 | 43 | F | Colgate | Mouthwash | T0 | PLD021t0 |
|  |  |  |  |  | T14 | PLD021t14 |
| PLD022 | 49 | F | Colgate | Mouthwash | T0 | PLD022t0 |
|  |  |  |  |  | T14 | PLD022t14 |
| PLD023 | 31 | M | Colgate | Mouthwash | T0 | PLD023t0 |
|  |  |  |  |  | T14 | PLD023t14 |
| PLD024 | 37 | M | Colgate | Placebo | T0 | PLD024t0 |
|  |  |  |  |  | T14 | PLD024t14 |
| PLD025 | 40 | F | Colgate | Placebo | T0 | PLD025t0 |
|  |  |  |  |  | T14 | PLD025t14 |
| PLD026 | 36 | M | Colgate | Placebo | T0 | PLD026t0 |
|  |  |  |  |  | T14 | PLD026t14 |
| PLD027 | 36 | F | Colgate | Mouthwash | T0 | PLD027t0 |
|  |  |  |  |  | T14 | PLD027t14 |
| PLD028 | 55 | F | Parodontax | Placebo | T0 | PLD028t0 |
|  |  |  |  |  | T14 | PLD028t14 |
| PLD029 | 37 | M | Licor del polo | Placebo | T0 | PLD029t0 |
|  |  |  |  |  | T14 | PLD029t14 |
| PLD030 | 32 | F | Colgate | Mouthwash | T0 | PLD030t0 |
|  |  |  |  |  | T14 | PLD030t14 |
| PLD031 | 22 | M | Colgate | Placebo | T0 | PLD031t0 |
|  |  |  |  |  | T14 | PLD031t14 |
| PLD032 | 34 | F | Gingilacer | Placebo | T0 | PLD032t0 |
|  |  |  |  |  | T14 | PLD032t14 |
| PLD033 | 29 | F | Sensodine | Mouthwash | T0 | PLD033t0 |
|  |  |  |  |  | T14 | PLD033t14 |
| PLD034 | 41 | F | Alipide | Placebo | T0 | PLD034t0 |
|  |  |  |  |  | T14 | PLD034t14 |
| PLD035 | 40 | F | Oral B | Mouthwash | T0 | PLD035t0 |
|  |  |  |  |  | T14 | PLD035t14 |
| PLD036 | 44 | M | Signal | Mouthwash | T0 | PLD036t0 |
|  |  |  |  |  | T14 | PLD036t14 |
| PLD037 | 24 | F | Colgate | Mouthwash | T0 | PLD037t0 |
|  |  |  |  |  | T14 | PLD037t14 |
| PLD038 | 21 | M | Colgate | Mouthwash | T0 | PLD038t0 |
|  |  |  |  |  | T14 | PLD038t14 |
| PLD039 | 40 | M | Marvis | Mouthwash | T0 | PLD039t0 |
|  |  |  |  |  | T14 | PLD039t14 |
| PLD040 | 41 | M | Colgate | Mouthwash | T0 | PLD040t0 |
|  |  |  |  |  | T14 | PLD040t14 |
| PLD041 | 42 | F | Colgate | Placebo | T0 | PLD041t0 |
|  |  |  |  |  | T14 | PLD041t14 |
| PLD042 | 57 | F | Colgate | Placebo | T0 | PLD042t0 |
|  |  |  |  |  | T14 | PLD042t14 |
| PLD043 | 44 | F | Colgate | Mouthwash | T0 | PLD043t0 |
|  |  |  |  |  | T14 | PLD043t14 |
| PLD044 | 34 | M | Colgate | Mouthwash | T0 | PLD044t0 |
|  |  |  |  |  | T14 | PLD044t14 |
| PLD045 | 28 | M | Colgate | Mouthwash | T0 | PLD045t0 |
|  |  |  |  |  | T14 | PLD045t14 |
| PLD046 | 35 | F | Colgate | Placebo | T0 | PLD046t0 |
|  |  |  |  |  | T14 | PLD046t14 |
| PLD047 | 27 | F | Colgate | Placebo | T0 | PLD047t0 |
|  |  |  |  |  | T14 | PLD047t14 |
| PLD048 | 32 | F | Deliplus | Placebo | T0 | PLD048t0 |
|  |  |  |  |  | T14 | PLD048t14 |
| PLD049 | 48 | F | Colgate | Mouthwash | T0 | PLD049t0 |
|  |  |  |  |  | T14 | PLD049t14 |
| PLD050 | 25 | F | Colgate | Mouthwash | T0 | PLD050t0 |
|  |  |  |  |  | T14 | PLD050t14 |
| PLD051 | 23 | M | Oral B | Placebo | T0 | PLD051t0 |
|  |  |  |  |  | T14 | PLD051t14 |
| PLD052 | 48 | F | Deliplus | Placebo | T0 | PLD052t0 |
|  |  |  |  |  | T14 | PLD052t14 |
| PLD053 | 31 | F | Colgate | Placebo | T0 | PLD053t0 |
|  |  |  |  |  | T14 | PLD053t14 |
| PLD054 | 23 | F | Colgate | Mouthwash | T0 | PLD054t0 |
|  |  |  |  |  | T14 | PLD054t14 |
| PLD055 | 50 | F | Colgate | Placebo | T0 | PLD055t0 |
|  |  |  |  |  | T14 | PLD055t14 |
| PLD056 | 31 | M | Colgate | Mouthwash | T0 | PLD056t0 |
|  |  |  |  |  | T14 | PLD056t14 |
| PLD057 | 61 | F | Colgate | Placebo | T0 | PLD057t0 |
|  |  |  |  |  | T14 | PLD057t14 |
| PLD058 | 27 | F | Deliplus | Placebo | T0 | PLD058t0 |
|  |  |  |  |  | T14 | PLD058t14 |
| PLD059 | 33 | F | Colgate | Mouthwash | T0 | PLD059t0 |
|  |  |  |  |  | T14 | PLD059t14 |
| PLD060 | 37 | F | Colgate | Placebo | T0 | PLD060t0 |
|  |  |  |  |  | T14 | PLD060t14 |
| PLD061 | 45 | F | Licor del polo | Placebo | T0 | PLD061t0 |
|  |  |  |  |  | T14 | PLD061t14 |
| PLD062 | 34 | F | Oral B | Placebo | T0 | PLD062t0 |
|  |  |  |  |  | T14 | PLD062t14 |
| PLD063 | 25 | M | Lacer | Mouthwash | T0 | PLD063t0 |
|  |  |  |  |  | T14 | PLD063t14 |
| PLD064 | 56 | F | Colgate | Placebo | T0 | PLD064t0 |
|  |  |  |  |  | T14 | PLD064t14 |
| PLD065 | 55 | F | Parodontax | Mouthwash | T0 | PLD065t0 |
|  |  |  |  |  | T14 | PLD065t14 |
| PLD066 | 27 | F | Signal | Mouthwash | T0 | PLD066t0 |
|  |  |  |  |  | T14 | PLD066t14 |
| PLD067 | 40 | F | Colgate | Mouthwash | T0 | PLD067t0 |
|  |  |  |  |  | T14 | PLD067t14 |
| PLD068 | 44 | F | Vitis | Mouthwash | T0 | PLD068t0 |
|  |  |  |  |  | T14 | PLD068t14 |
| PLD069 | 30 | F | Oral B | Placebo | T0 | PLD069t0 |
|  |  |  |  |  | T14 | PLD069t14 |
| PLD070 | 55 | F | Colgate | Placebo | T0 | PLD070t0 |
|  |  |  |  |  | T14 | PLD070t14 |
| PLD071 | 48 | M | Colgate | Placebo | T0 | PLD071t0 |
|  |  |  |  |  | T14 | PLD071t14 |
| PLD072 | 26 | F | Colgate | Mouthwash | T0 | PLD072t0 |
|  |  |  |  |  | T14 | PLD072t14 |
| PLD073 | 50 | M | Deliplus | Placebo | T0 | PLD073t0 |
|  |  |  |  |  | T14 | PLD073t14 |
| PLD074 | 30 | F | Colgate | Mouthwash | T0 | PLD074t0 |
|  |  |  |  |  | T14 | PLD074t14 |
| PLD076 | 20 | F | Colgate | Placebo | T0 | PLD076t0 |
|  |  |  |  |  | T14 | PLD076t14 |
| PLD077 | 40 | M | Vitis | Placebo | T0 | PLD077t0 |
|  |  |  |  |  | T14 | PLD077t14 |
| PLD078 | 26 | F | Colgate | Mouthwash | T0 | PLD078t0 |
|  |  |  |  |  | T14 | PLD078t14 |
| PLD079 | 55 | F | Colgate | Placebo | T0 | PLD079t0 |
|  |  |  |  |  | T14 | PLD079t14 |
| PLD080 | 25 | F | Oral B | Placebo | T0 | PLD080t0* |
|  |  |  |  |  | T14 | PLD080t14* |
| PLD081 | 25 | F | Deliplus | Placebo | T0 | PLD081t0 |
|  |  |  |  |  | T14 | PLD081t14 |
| PLD082 | 25 | M | Colgate | Mouthwash | T0 | PLD082t0 |
|  |  |  |  |  | T14 | PLD082t14 |
| PLD083 | 35 | F | Parodontax | Mouthwash | T0 | PLD083t0 |
|  |  |  |  |  | T14 | PLD083t14 |
| PLD084 | 24 | M | Colgate | Mouthwash | T0 | PLD084t0 |
|  |  |  |  |  | T14 | PLD084t14 |
| PLD085 | 19 | F | Colgate | Mouthwash | T0 | PLD085t0 |
|  |  |  |  |  | T14 | PLD085t14 |
| PLD086 | 34 | M | Colgate | Mouthwash | T0 | PLD086t0 |
|  |  |  |  |  | T14 | PLD086t14 |
| PLD087 | 28 | F | Colgate | Placebo | T0 | PLD087t0 |
|  |  |  |  |  | T14 | PLD087t14 |
| PLD088 | 23 | F | Colgate | Placebo | T0 | PLD088t0 |
|  |  |  |  |  | T14 | PLD088t14 |
| PLD090 | 22 | F | Binaca | Mouthwash | T0 | PLD090t0 |
|  |  |  |  |  | T14 | PLD090t14 |
| PLD091 | 36 | F | Colgate | Mouthwash | T0 | PLD091t0 |
|  |  |  |  |  | T14 | PLD091t14 |
| PLD093 | 34 | F | Colgate | Placebo | T0 | PLD093t0 |
|  |  |  |  |  | T14 | PLD093t14 |
| PLD094 | 50 | F | Colgate | Mouthwash | T0 | PLD094t0 |
|  |  |  |  |  | T14 | PLD094t14 |
| PLD095 | 36 | F | Sensodine | Placebo | T0 | PLD095t0 |
|  |  |  |  |  | T14 | PLD095t14 |
| PLD096 | 19 | F | Colgate | Mouthwash | T0 | PLD096t0 |
|  |  |  |  |  | T14 | PLD096t14 |
| PLD097 | 31 | F | Colgate | Placebo | T0 | PLD097t0 |
|  |  |  |  |  | T14 | PLD097t14 |
| PLD098 | 33 | M | Colgate | Placebo | T0 | PLD098t0 |
|  |  |  |  |  | T14 | PLD098t14 |
| PLD099 | 25 | F | Colgate | Placebo | T0 | PLD099t0 |
|  |  |  |  |  | T14 | PLD099t14 |
| PLD100 | 23 | F | Colgate | Mouthwash | T0 | PLD100t0 |
|  |  |  |  |  | T14 | PLD100t14 |
| PLD101 | 23 | M | Colgate | Placebo | T0 | PLD101t0 |
|  |  |  |  |  | T14 | PLD101t14 |
| PLD102 | 20 | F | Colgate | Mouthwash | T0 | PLD102t0 |
|  |  |  |  |  | T14 | PLD102t14 |
| PLD103 | 66 | M | Colgate | Mouthwash | T0 | PLD103t0 |
|  |  |  |  |  | T14 | PLD103t14 |
| PLD104 | 20 | F | Colgate | Placebo | T0 | PLD104t0 |
|  |  |  |  |  | T14 | PLD104t14 |
| PLD105 | 30 | M | Colgate | Placebo | T0 | PLD105t0 |
|  |  |  |  |  | T14 | PLD105t14 |

* Due to the low sequence counts, these samples were excluded for further paired data analysis.

Table S2. Formulation of the mouthwash used to conduct the study. Both mouthwash and placebo (mineral water) were packaged in opaque bottles to minimise possible volunteer bias. As the mouthwash is commercially available, the concentrations of the compounds are not specified.

| Mouthwash Formulation |
| --- |
| Xylitol Hydroxyacetophenone Sodium Citrate Poloxamer 407 Sodium Gluconate Zinc Chloride O-Cymen-5-Ol Aroma Sodium Fluoride Dipotassium Glycyrrhizate Menthol Sucralose Mentha Piperita Oil Citric Acid Neohesperidin Dihydrochalcone Vanillin CI 42090 CI 47005 |

Table S3. Bacterial genera showing significant changes in at least one of the two treatments (placebo and mouthwash) at the end of the study, with their respective mean relative abundance and standard error, the p value obtained from the Wilcoxon signed-rank test and the corresponding statistical significance (NS, not significant; *, p value <0.05; **, p value <0.01; *** p value <0.001). In addition, the table shows the trends observed within each group and genus, indicating the percentage of samples that showed an increase, decrease or no change in relative abundance after treatment.
